# Out-of-Pocket Payments, Health Care Access and Utilisation in South-Eastern Nigeria: A Gender Perspective

**DOI:** 10.1371/journal.pone.0093887

**Published:** 2014-04-11

**Authors:** Michael N. Onah, Veloshnee Govender

**Affiliations:** 1 STRIVE Research Consortium, Wits Reproductive Health and HIV Institute (Wits RHI), Faculty of Health Sciences, University of Witwatersrand, Johannesburg, South Africa; 2 Health Economics Unit (HEU), School of Public Health, Faculty of Health Sciences, University of Cape Town, Cape Town, South Africa; University of Oxford, Kenya

## Abstract

Out-of-pocket (OOP) payments have severe consequences for health care access and utilisation and are especially catastrophic for the poor. Although women comprise the majority of the poor in Nigeria and globally, the implications of OOP payments for health care access from a gender perspective have received little attention. This study seeks to fill this gap by using a combination of quantitative and qualitative analysis to investigate the gendered impact of OOPs on healthcare utilisation in south-eastern Nigeria. 411 households were surveyed and six single-sex Focus Group Discussions conducted. This study confirmed the socioeconomic and demographic vulnerability of female-headed households (FHHs), which contributed to gender-based inter-household differences in healthcare access, cost burden, choices of healthcare providers, methods of funding healthcare and coping strategies. FHHs had higher cost burdens from seeking care and untreated morbidity than male-headed households (MHHs) with affordability as a reason for not seeking care. There is also a high utilisation of patent medicine vendors (PMVs) by both households (PMVs are drug vendors that are unregulated, likely to offer very low-quality treatment and do not have trained personnel). OOP payment was predominantly the means of healthcare payment for both households, and households spoke of the difficulties associated with repaying health-related debt with implications for the medical poverty trap. It is recommended that the removal of user fees, introduction of prepayment schemes, and regulating PMVs be considered to improve access and provide protection against debt for FHHs and MHHs. The vulnerability of widows is of special concern and efforts to improve their healthcare access and broader efforts to empower should be encouraged for them and other poor households.

## Introduction

### Gender, Out-of-Pocket Payments and Health Care Access

A key message of the World Health Report 2010 is that “…millions of people cannot use health services because they have to pay for them at the time they receive them. And many of those who do use services suffer financial hardship, or are even impoverished, because they have to pay.”([1] p:113). To date, much of the focus has been on the implications of out-of-pocket payments (OOPs), including user fees for individuals and households in relation to socio-economic status [2], [3]. While this is clearly important and warranted, other researchers have been pointing to the barriers that other vulnerable groups (i.e., women, children, ethnic minorities) face [4]–[8]. Considering that women represent 70 per cent of the world’s poor [9], the influence of gender on access in the context of out-of-pocket payments is important. Research has shown important differentials in financial access between men and women. For example, “women incur more out-of pocket expenditure than men”, “paying for health care and other reproductive health services places a high financial burden on women” and “out-of-pocket expenditure may prevent more women than men from utilising essential health services”([10] p:650).

Research on gender and health care access has also broadened to consider implications for access from the perspective of female-headed households (FHHs). This has been prompted by the growing number of FHHs globally [11]. In 1998, almost a fifth of households worldwide and in sub-Saharan Africa was female-headed [12]. In both developed and developing countries, studies have revealed that FHHs are likely to have different demographic, sociological, and economic characteristics from MHHs and that these differences have major implications for health care access and utilisation [13], [14].While data are inconclusive on whether FHHs are poorer than their male counterparts [15], data from across different settings suggest that they have higher dependency ratios and are typically headed by older women, who are often widows [9]. Research from Ghana indicated that widows and single women are especially vulnerable and that particularly those from poor households found direct costs of care an access barrier [16].

### Nigeria

Since the fall in oil prices in Nigeria in the 1980s, economic growth has slowed, with adverse implications for government budgetary allocations towards health care and other social sectors [17]. In 2010, it was found that the share of government expenditure on health care was merely 3.5%; this is considerably below the 2001 Abuja commitment which called on all signatory governments (including Nigeria) to allocate 15% of government expenditure to health care [18]. In Nigeria, public spending per capita for health is less than USD 5 and can be as low as USD 2 in some parts; a far cry from the USD 34 recommended by WHO for LMICs [1]. Private health expenditure as a percentage of total health expenditure is almost 64%. Households contribute almost 96% of total health care expenditure through OOP payments [1]. This is important in the context that 34.1% of the population lives below the poverty line (i.e. less than USD1 per day) [19]. Clearly, the burden of paying for health care is especially regressive for poor households.

In Nigeria formal and informal user fees are charged in health care facilities with fees differing according to the type of care sought and the level of facility utilised [20]. The under-resourcing, poor provision and delivery of public health services and the burden of user fees for roughly every treatment item has encouraged the growth of and demand for private health care [20]. Private health care accounts for almost 66 per cent of total health care in Nigeria [18] and covers a wide range of providers, including patent medicine vendors (PMVs), pharmacy shops, traditional medicine sellers, maternity homes, clinics, and private tertiary hospitals, many of which are unregulated (e.g. PMVs).

Women lag behind men in education and employment. Women have lower levels of literacy compared to men (44% vs. 67%) [21]. This has implications for the type of employment opportunities that women have. Data from the NBS (2009) show that women had a higher unemployment rate (42%) compared to men (22%), 55% of the employed were low-grade staff in the formal sector and those employed in the farming sector were predominantly employed as unpaid (family) labour. In the rural communities, controls of income from farm proceeds are in the hands of men [22]. A household survey concluded that utilisation of health care by women is mediated by their role in decision making and resource allocation within households [21]. Results from the same survey found that a woman is more likely to be a part of the decision-making process on how her earnings and her husband’s earnings are spent if she earns more than or the same amount of money as her husband. The south- east zone where the study was located had the lowest percentage of women making sole decisions regarding their earnings (27%). Clearly, lack of access to paid employment and inequitable decision-making power within especially poor households might mean that when poor women are confronted with OOP costs for health care, it can delay or deter utilisation [23].

Studies from Nigeria have neglected the issue of affordability in the context of OOP payment for male-and female- headed households. Previous research has either analysed the effects of OOP payment on the poor or on female specific health services [22], [24]–[26]. Considering that women lag behind in education and employment in Nigeria and knowing the impact of lack of education on employment opportunities and to a great extent; income generation, the importance of a gendered study of OOP payment and affordability becomes necessary. Thus, this study seeks to investigate and fill this gap by investigating through a combination of quantitative and qualitative analysis the impact of OOPs on health care access on male-and female-headed households.

## Methods

### Study area

This study was conducted in Nsukka Local Government Area (NLGA), located in the northern part of Enugu State in south-eastern Nigeria. NLGA comprises 1 urban and 14 rural communities, with a population of almost 310,000, comprising approximately 63, 705 households [27]. Agriculture is the main economic activity and the area is predominantly Ibo (i.e., ethnic group) who are mainly Christians with a few traditional believers in the rural areas. Like other parts of Nigeria, women, including FHHs in NLGA, are less educated, engage more in low level subsistence farming and largely employed in informal employments with low income generation abilities [28]. Heads of FHHs are largely older than their MHHs counterpart and headship is mainly as a result of widowhood [29].

The urban community is a university town and has a wider variety of health care providers including public and private hospitals, primary health care providers, PMVs, and pharmacies. In the rural communities, primary health facilities referred to as health centres and PMVs are the main health services providers. If hospital care is required, people travel to the urban hospital which is between 18– 30 kilometres away. All government facilities charge user fees, although charges differ depending on the type of care sought and patients also pay for drugs. There are exemptions for HIV treatment, leprosy and maternal health.

### Sampling and data collection

The study used both cross-sectional household surveys and focus-group discussions (FGDs) methods to investigate the research questions. A total of 411 households were interviewed (111 in urban and 300 in rural communities). A household is designated as comprising individuals who live in the same house and who have common arrangements for basic domestic and/or reproductive activities such as cooking and eating” ([30] p:22). Household surveys were chosen over facility-based survey since an understanding of access requires considering the views and experiences of both users and non-users of health care.

The following approach was adopted in order to determine the sample size. Given that in 2010, NLGA comprised 69,705 households, the sample size for this study was calculated using Taro Yamane (1967) specification (see Ataguba et al. 2008 [29]) given as:
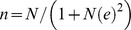
where; *n* = sample size to be estimated, *N* = population size, and *e* = error margin at 95% confidence interval. The population and number of households were extrapolated based on the 2006 population census and an annual 3% population growth rate [28]

The minimum sample size required to obtain a confidence interval of 5% around this figure was 400 households. The sample size was increased to 411 households to allow for data incomplete questionnaires.

A multi-stage sampling method was used to select households for the survey and the urban and 14 rural communities were classified into enumeration areas (EAs) [29]. First, to ensure adequate representation of both urban and rural EAs, NLGA was stratified into urban and rural communities, representing 30% and 70% of the population respectively. A total of 24 EAs were selected (3 urban, 21 rural) based on probability-proportional to size (PPS) and 39 and 21 households were sampled in each of the urban and rural EAs respectively. In the second stage, a simple systematic random sampling method was used to identify survey households from each of the EAs. The sample of households was appropriately weighted in analysis using the inverse probability weighting method which denotes the inverse of the probability that the observation is included in the analysis due to the chosen sample design [29]. Under the method, each household selected from each enumeration area (EA) is weighted to make it representative of the entire EA such that the sum of the weights for each EA should equal the approximate number of households in that EA. The questionnaire was administered to preferably the household head or the spouse and in their absence, a senior household member. The interviews were conducted by9 trained field workers.

Six single-sex FGDs (2 urban, 4 rural) were conducted in 3 communities (1 urban, 2 rural). Each FGD consisted of 8 to 11 participants. Single-sex interviews were considered appropriate given the focus of the research on gender, health care access, coping strategies and intra-household decision-making and sensitive issues which are likely to be spoken of more freely and without fear of reproach in a single-sex group. FGDs were organised to ensure that participants were of similar economic background and economic activity (traders, teachers, farmers, women religious and trading groups), besides considerations of gender. Invitations were sent to men and women in advance of their meeting days. All participants were 18 years and older. The discussions were conducted in the village square and community centres. FGDs were audio taped, transcribed and translated into English and the transcripts were thematically coded and analysed.

### Study variables and data analysis

The household surveys investigated households’ socio-economic and demographic status, general household and health care expenditure patterns, household assets, utilisation patterns, healthcare financing, intra-household decision making and coping strategies. The questionnaire was adapted from an earlier survey conducted in the same region [17] and was translated into the local language.

While the household survey provided important data for quantifying the differences and similarities in utilisation patterns between male-and female-headed households, it was inadequate in helping us understand why these differences existed. In this study, the gap was filled through the use of FGDs, which aimed to provide more qualitative data around issues of the burden of OOPs and its implications for health care access, coping strategies, household decision-making in general and more specifically around health expenditure from the perspective of men and women. The FGDs were taped-recorded and notes were taken which were then transcribed. The transcripts were read and broad themes relevant to the study objectives were extracted. In addition, new themes which were identified during the review of the transcripts were also captured and presented in the results.

The quantitative data were inputted and managed using EpiData software and then exported to STATA software for analysis. Associations between quantitative variables were assessed using the Chi Square test. A bivariate analysis was conducted and variables which were significant at a probability value (*p*-value) equal to or less than 0.05 were selected and included. The bivariate analysis was specified to examine the associations between the sex of the household head and other variables including utilisation, decision- making relating to general and health care expenditure, insurance ownership, health care payment options, health status, reasons for not seeking care and coping strategies. Options were subdivided into dichotomous responses of “0” for no and “1” for yes.

The monthly cost of health care was calculated by the summation of direct costs (i.e., registration/card fees, consultation fees, laboratory tests and drug costs) that a household incurred in the month previous to the interview. This cost was converted to United States Dollar (2010 exchange rate of US$1.00  = 150 naira).

This study used asset indexes as a measure of socio-economic status of households. An asset index was chosen over other measures for constructing the socio-economic status of households. This is because it is easier to collect asset data in contexts like the study site, and income and expenditure data would also not fully represent the household socio-economic status [31], [32]. Information on ownership of electronic equipment (e.g. radio, television and fridge), transport (bicycle, motorcycle and motorcar), sources of energy (kerosene lamp, electricity generators and rechargeable lamps) were pooled together to construct the index. In conducting the principal component analysis, the first component factor was used to represent the asset index. On this basis, the study population was classified into four quartiles (i.e., least poor, poor, very poor and poorest).The first component factor is defined statistically as a weighted sum of the various assets used to assess household wealth, in order for that component to explain as much as possible of the variance observed in asset ownership between households.

To estimate the proportion of households incurring potentially catastrophic burdens, costs incurred by each household for health care were divided by household monthly expenditure and reported as a percentage. The household total expenditure was derived by annualising household weekly expenditure on food and beverages and household monthly living expenditure on items such as rent, if any, energy and clothing. The total annual expenditure was then divided by 12 to arrive at the household’s monthly expenditure. health care expenditures are deemed catastrophic if the expenditure is 10% or more of household income [33], where catastrophic implies that such expenditure levels are “ likely to force households to cut their consumption of other minimum needs, trigger productive asset sales or high levels of debt and lead to impoverishment”([34] p:149).

### Ethics Statement

The study received ethical approval from the University of Cape Town Ethics Committee and permission was also sought from Nsukka LGA authorities. Informed consent (oral and written) was obtained from all respondents in the household surveys and participants in the FGDs. Oral consent for the FGDs were conducted in the first language of the participants and were captured using an audio recorder, while written consents were used for the household survey and were captured as part of the questionnaires. Oral consent was used in the FGDs due to the difficulties experienced during the household survey on respondents’ literacy level; however participants signed an attendance register. The consent forms were in English and the local language and were read out to obtain oral consent for the FGDs. The consent forms, interview guides, questionnaires, and consent procedures were part of the ethical submissions that were approved for the study. Household interviews and FGDs were conducted in the first language of the respondents and participants.

## Results

### Demographic and socio-economic characteristics

Almost 40% of households were FHHs (Table 1). On average, the heads of FHHs were older (57 years, compared to 48 years for MHHs [CI 47.94–58.07]), more likely to have no schooling, (56.0% compared to 16.9% in MHHS), more likely to be widowed (82.3% vs. 8.7%), have smaller households (2.0 vs. 4.0), less likely to have health insurance (4.5% vs. 15.1%) and more likely to be located in the poorest quintile (64.0% vs. 39.1% in MHHs). Moreover, they were also more likely to be engaged as subsistence farmers (69.8% vs. 49.8%).

**Table 1:** Demographic and Socio-Economic characteristics of household heads.

Demographic factors	Variable	Sex of household head		Significance (*p*-value)
		Female(n = 160)	Male(n = 251)	
	Average age of household head (years)	57	48	0.00
	Education level of household head			
	None	56.0	16.9	
	Secondary education	43.9	59.2	
	Post-secondary education	0.0	23.9	0.00
	Marital status			
	Never married/divorced	15.6	6.8	
	Living with spouse	1.3	84.5	
	Widowed	82.5	8.7	0.00
	Household size (average)	2.0	4.0	0.00
	Location			
	Urban (%)	30	25.1	0.03
Socioeconomic factors	Insured Household (%)	4.5	15.1	0.00
	Asset index*			
	Poorest	64.0	39.1	
	Poor	2.5	6.0	
	Rich	20.7	26.6	
	Richest	12.9	28.3	0.00
	Employment status of household head			
	Unemployed/pensioner	7.5	7.9	
	Petty trading/hawking	8.7	8.7	
	Formally employed (private/public sector)	1.8	16.7	
	Self-employed (artisans)	6.2	8.7	
	Farmer (subsistence)	69.3	49.8	
	Trader	6.2	7.9	0.00

^*^1^st^ component accounted for 47% of the total variation in the PCA

### Perceptions of illness in the context of poverty

Although the focus of the study surrounded questions on OOP and access, the FGDs brought up a range of issues that went beyond access and OOP issues. Two of these issues which emerged were how men and women spoke of illness in the context of poverty which has important implications on treatment seeking behaviour. In relation to health and ill-health, both men and women in the FGDs spoke of women’s vulnerability to illness with implications for treatment seeking behaviour:

*Women are more inclined to illness, thus making their health care costly. My wife always falls sick from even simple cold and so I spend too much on her health*- 29 year old male (rural).

In some instances, women and men spoke movingly of poverty, the demands of physical labour and family responsibility as key factors underlying their vulnerability to ill-health:

*Poverty is the major cause of illness. Because of no money women don’t eat well and become sick* – 24 year old widow (rural).

*Female health care is more expensive to treat than male’s. You know we are weaker by nature but these days we even do men’s work and are more exposed to illness *– 20 year old female (urban) (MHH).

*The dynamics of providing for your family can affect your health. I had to work extra when my children got into secondary school, providing for their school fees and feeding them. They started eating more as growing children. The stress got me sick most times *– 69 year old male (urban).

### Treatment seeking behaviour

A higher percentage of members in FHHs (32.4%) reported being sick in the previous month compared to MHHs (25.2%) (*p<0.05*). Within FHHs, 41% of those that reported sickness in the past month were between the ages of 1and16 years, while 4% were adults between the age of 18 and 25 years. Also 45% of household heads in FHHs reported illness. In MHHs, 75% of those that reported illness were between the age of 1 and 16 years, 19% were adults and 6% were household heads.

**Figure 1:**
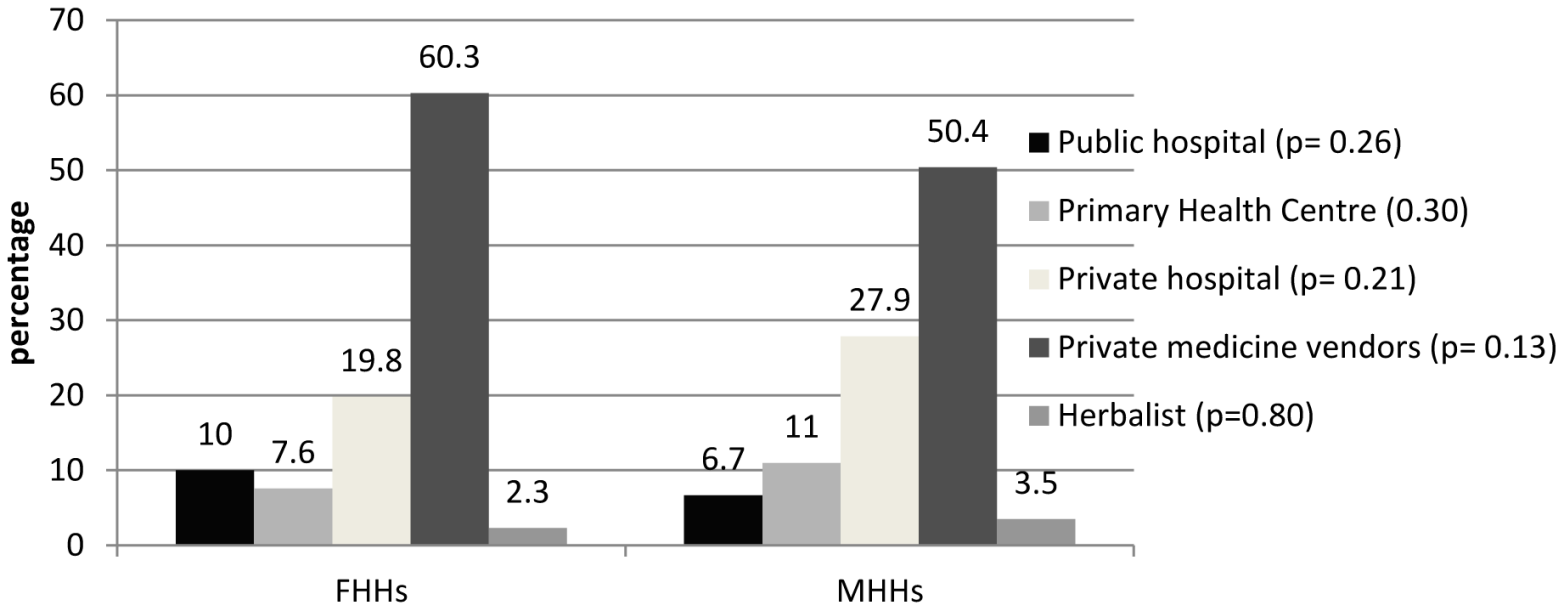
Type of health care provider utilized.

There were also differences in utilisation of health services between FHHs and MHHs (Figure 1). While PMVs were the single most popular health care provider for both FHHs and MHHs, a higher percentage (60.3%) of members in FHHs utilised PMVs compared to MHHs (50.4%). MHHs utilised more private hospitals and primary health care centres (25.9%, 11% vs. 19.9% and 7.6% in FHHs respectively). These findings were not statistically significant.

Qualitative findings also indicate that health-seeking behaviour differed between men and women and also between the rural and urban areas. In the urban area, participants reported using private hospitals and public hospitals for consultations. They used PMVs for drug purchases:

*My family uses the private hospital close to our house when we are sick and then buy the drugs from the chemist (PMV) around the corner. But these days the hospital now insists we buy drugs there. There is a big problem because they are too expensive*- 49 year old male (urban).

*I use the government hospital when I am sick but due to the long queues there, I go very early in the morning and spend the whole day there*- 20 year old single mother (urban).

Rural male and female participants sought care more often from PMVs than from the primary health centres, mainly because of poor perceptions of quality of care associated with the latter. In the rural areas, many of the PMVs are owned and operated by midwives or nurses and are often the preferred provider in the case of a minor illness:

*The health centre here cannot even give you good drugs for simple malaria. We have to pay more at the nurse’s shop (PMV) to buy good drugs when we are sick* - 59 year old widow (rural).

In the event of a serious illness, including in-patient care and deliveries, the only option is to travel to the urban area:

*I only use private hospitals now because they are value for money no matter the distance to get there. The last time my son was sick, we waited for hours for a doctor at the health centre....my son nearly died*- 49 year old female (rural).

OOP payment was a major source of funding health care expenditure for both MMHs and FHH (See Table 2). MHHs reported a relatively high percentage of OOPs as a payment option for health care than FHHs. This was not statistically significant. In addition, FHHs reported more making in-kind payment and paying in instalments while MHHs in comparison reported higher levels of prepayment (i.e. insurance).

**Table 2:** Health care payment options.

		Sex of household head		
	Household members	Female (n = 398)	Male (n = 1117)	*p-*value
**Payment options**	OOP payment	86.9	91.8	0.12
	Health insurance	3.9	14.7	0.00
	Instalment	20.8	19.3	0.74
	In-kind	16.2	7.9	0.01

Also, findings from the FGDs show that payment options for households in rural areas differed from those in the urban area. Rural areas reported instalment payments and payment in-kind whereas households in urban areas reported medical insurance coverage and making OOP payments.

*We traders pay in cash when we go to the hospital. Nobody will even talk to you if you want to owe them while they treat you*- 44 year old male (urban)

*The doctor here is very good to us. He can treat you while you pay back as little as you can. Sometimes he even takes our game meat as payment*- 29 year old female (rural) (MHHs) and reported by many households in the rural setting including FHHs

*Since my wife got this government work, we can now go to the hospital and not worry about having cash in hand. She has this new National Health Insurance*- 49 year old male (urban).

### Burden of Out-Of-Pocket payment and untreated morbidity

To understand the cost burden of health care expenditure on households, monthly health care costs as a percentage of household monthly expenditure was examined across households and by sex of the household head and by socioeconomic group. Untreated morbidity was as also measured by sex of household head and socioeconomic group.

**Figure 2:**
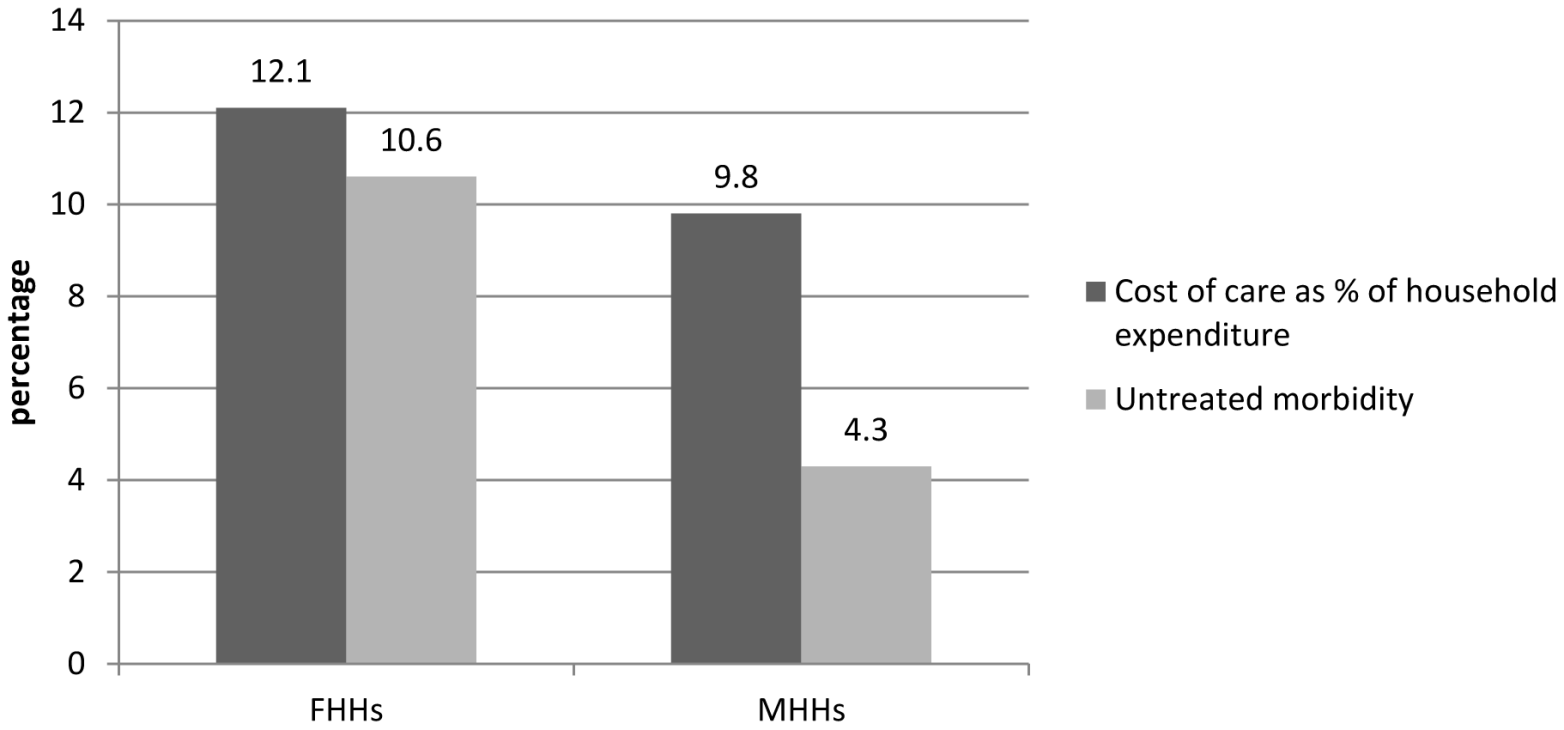
Cost burden and untreated morbidity (*p<0.05*).

Households on the average spent $33 monthly on health care (CI: 29.71–35.66; median: $30; inter-quartile range: $23–$35). On the average, MHHs spent more on health care than FHHs ($32.2 vs. $24.6 [CI: 23.89–33.04]). But when cost is viewed as a percentage of households monthly expenditure, FHHs spent about 12.1% of their total monthly expenditure on health (9.8% for MHHs) (Figure 2). In line with this, FHHs reported higher levels of being sick and not seeking care (10.6%) relative to MHHs (4.3%). When cost burden and untreated morbidity is disaggregated by socioeconomic group (See Table 3), the poorest households incurred the highest cost burdens (14.8% and 12.4%) irrespective of the sex of the household head and households that reported untreated morbidity were concentrated more within the group (65.4% and 57.1%). On the other hand, the least poor FHHs spent as little as 5.2% and 2.1% for MHHs on health care costs and households within this group that reported untreated morbidity was as low as 3.7% for FHHs and 2.1% for MHHs.

**Table 3:** Distribution of health care costs burden and untreated morbidity across socioeconomic groups.

		MHHs		FHHs	
Socioeconomic group		Cost burden*	Untreated morbidity*	Cost burden*	Untreated morbidity*
	Poorest	12.4	57.1	14.8	65.4
	Poor	10.2	31.8	13.1	25.8
	Rich	6.6	9.1	7.4	5.1
	Richest	2.1	2.0	4.2	3.7

* Indicates significance at p<0.05

**Figure 3:**
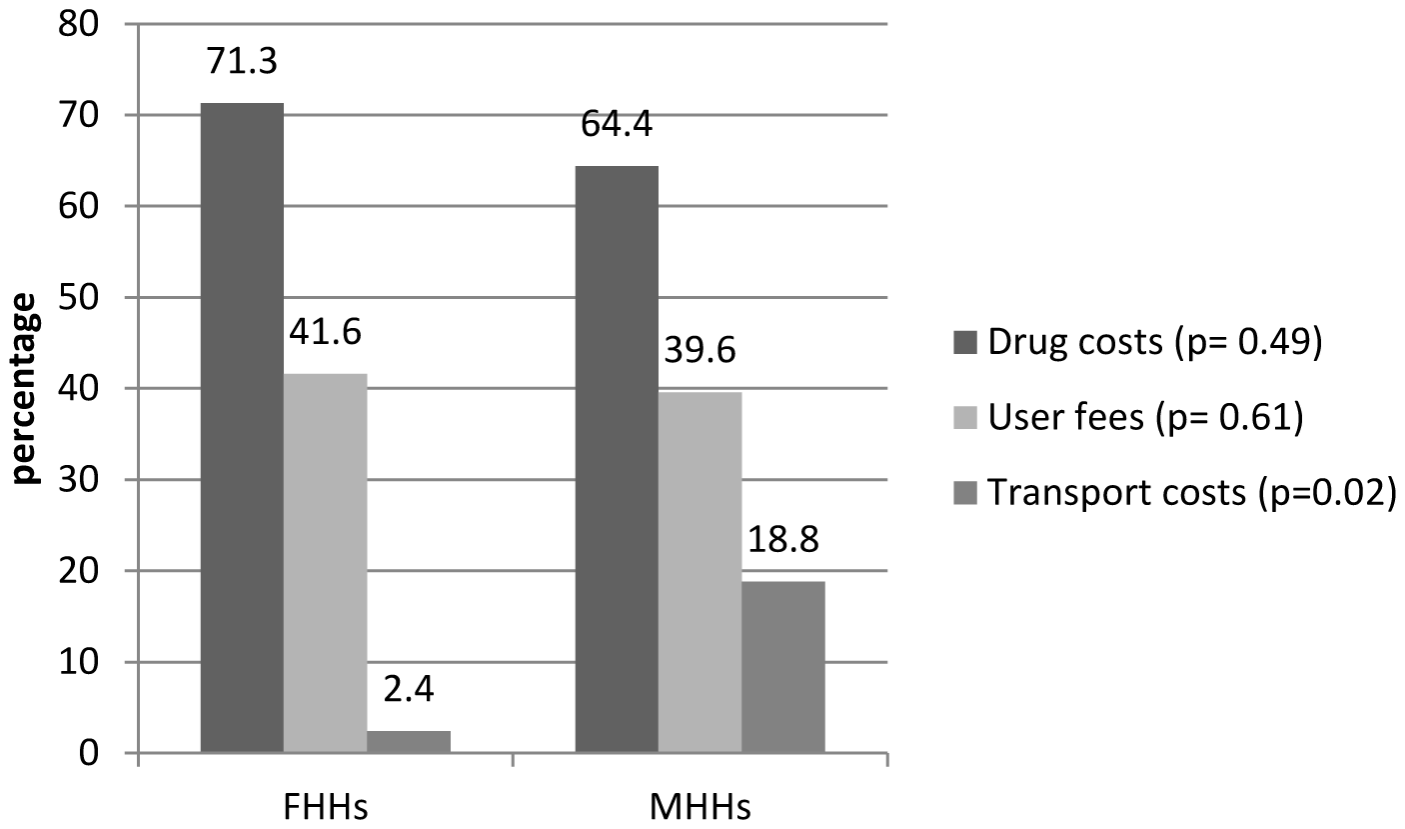
Affordability reasons for not seeking care.

For FHHs, the most important reasons for not seeking care were drug costs and user fees as over half of the sick members gave these as reasons (Figure 3). MHHs also reported high percentages (64.6%) of drug cost and user fees (41.6%) as a major reason for not seeking care. Reports of barriers of drug costs and user fees were higher in FHHs (71.3% and 39.6% respectively) than in MHHs, while MHHs reported higher levels of transport (18.8%) costs as a barrier to seeking care. Transport cost was significant while other costs were not.

Of importance is the report of untreated morbidity in the FGDs, in the context of affordability with focus on costs, both MHHs and FHHs spoke of ways of coping with what they considered to be non-severe illness.

*Some sickness goes on their own so there is no need of wasting money on drugs*- 50 year old male (urban)

*Going to the clinic is too expensive these days so if I have minor illness, I eat fruits and vegetables and hope it goes*- 59 year old widow (rural)

Also quotes from the FGDs highlight the significance of travel costs and drug costs for those in the rural areas requiring care. This falls in line with the availability and perceptions of primary health centres. This inspires the utilisation of private hospitals and PMVs and has implications on affordability as utilisation incurs higher cost:

*The health centres here are not functioning so I had to pay a lot of money to hire a car to take my wife to the town when she wanted to deliver in the middle of the night* - 55 year old male (rural)

*The health centre here cannot even give you good drugs for simple malaria. We have to pay more at the nurse’s shop (PMV) to buy good drugs when we are sick* - 59 year old widow (rural).

### Household coping strategies

In the event of illness, drawing from savings was reported by 80.0% and 90.4% of members of MHHs and FHHs respectively (see Table 4). These savings include cash and other durable food items stored as emergency funds as detailed further in the FGDs. This is followed by ‘someone else paying’, which can come in the form of gifts or loans for repayments. For FHHs after savings, the most important means of funding health care expenditure is ‘someone else paying’. Subsidies for health care were reported more in MHHs. This can be related to the higher percentage of ownership of medical insurance among MHHs. Borrowing, exemptions and group contributions were not significant.

**Table 4:** Household coping strategies.

		Sex of household head		
		Female (n = 398)	Male (n = 1117)	*p*-value
**Coping strategies**	Drew on savings	80.0	90.4	0.00
	Borrowed money	7.7	8.2	0.85
	Paid by non-household member	22.3	14.0	0.03
	Exempted from payment	3.9	4.3	0.83
	Payment was subsidized (insured)	2.3	12.6	0.00
	Contributed to group scheme *(isusu)*	6.9	8.6	0.55

Qualitatively, FHHs and MHHs identified and discussed a wide range of strategies that they employed when faced with health care costs. Funds for paying for health care were obtained from different sources which ranged from the most preferred (i.e., drawing from savings and sale of assets) to the least preferred (i.e., borrowing from a money lender or group contributions (i.e., *isusu*):

*We get money from farming and sales of stored goods that are seasonal; we buy palm oil and honey at cheap prices during the period of plenty and sell them when we need urgent money *– 49 year old female (rural) (MHHs). This was also a general claim from female participants in the rural areas including FHHs

*We also borrow from “interest people” (money lenders) but it is the worst due to the possibility of losing your collateral and the high interest they charge *– 59 year-old male (urban). This claim was supported by other members in the FGD

As illustrated by the first quotation, savings do not only refer to money but also to the deliberate strategy of buying essential household items not for the purposes of present or future consumption but as source of emergency funds. Following drawing on savings, MHHs in both urban and rural areas spoke of selling off assets:

*We usually have goats, stored food like yam, and fowls in the house. If there is no money, we sell them and use the money to pay for care* – 69 year old males (rural). This was a common strategy reported by males in the FGDs

*I sold my land when my wife and son had food poisoning*- 29 year old male (urban).

*We sold the cassava on our land for money when my husband was very sick. Although you don’t get much from such sale, it is better than nothing*- 40 year old female (rural) (MHHs).

If households still required money, following the sale of assets, they would then turn to borrowing from friends and relatives. Also, having friends or relatives borrow on their behalf was reported by male participants:

*We borrow from friends or relatives and if they don’t have, they can borrow on our behalf *– 65 year old male (rural).

*I borrow from my extended family when my household runs short of money* – 29 year old female (urban) (FHHs).

*We borrow from the meeting (associations/group contribution (isusu)) that we belong to* – 60 year old woman (rural). This claim was also supported by other rural women including those in FHHs

In what appears to be an exhaustion of options and a final action of desperation, a single mother sold her clothes to pay for her son’s medical care.

*I sold my wrappers (clothes) to pay for medical care of my son when he had hepatitis *– 49 year old single mother (urban).

### Coping strategies for re-paying health-related debts

Both men and women reported that in the event of debt arising from health care payments, a number of coping strategies are employed by households. Male participants reported increasing farming activities in order to generate more revenue to pay back the loans:

*I increase farming activities to enable me raise enough money to pay off the debt. I increase the portion of land I farm to get more money *– 65 year old male (rural).

Going to or sending family to work for those the money was borrowed from was often reported by men and women in both urban and rural areas. Some also suggested sending their relatives to work for those they owe as a means of clearing debt:

*I go and work in the farm of the person I owe as a means of payoff *– 56 year old male (rural).

*Borrowing from loan shark (money lenders) is very difficult and it is the last resort because of the high interest rate they charge; so you can send your children to work on other farms for wages to enable you fast track the payment* – 59 year old male (urban).

*I sent my brother to work for the person I borrowed money from* – 49 year old widow (rural).

*I used my motorcycle to borrow money from the market association when my husband was sick*- 39 year old female trader (urban).

Widows, without an asset base and limited options to draw on reported resorting to hard manual labour to generate an income and cutting down on food as strategies towards settling the debt:

*We sweep the bushes for pebbles that I sell to those building houses to enable me pay for the money I borrowed* – 24 and 59 year old widows (rural).

*I had to cut down on the food we eat in my house because we had debts to pay…I joined in carrying blocks for those building houses for wages to help me pay the debt* – 20 year old widow (rural).

## Discussion and Conclusion

This study has confirmed the vulnerability of FHHs as indicated by a range of demographic (widows, older, less educated) and socio-economic (predominantly poor and employed as subsistence farmers) factors, which contribute to gender-based household differences in health care access, cost burden, choices of health care providers, methods of funding health care and coping strategies.

Although a higher percentage of FHHs reported ill health when compared to MHHs, suggesting a greater need for health care, they reported lower levels of utilisation suggesting access barriers, particularly those relating to affordability. Although only transport cost was statistically significant in the survey, qualitatively, affordability reasons were largely mentioned as a major cost for seeking care. While this was not statistically significant, qualitatively, OOP payments were generally the main source of funding for health care, placing a heavy cost burden on households and were found to be regressive and catastrophic for the two poorest quartiles across all households and MHHs and FHHs as defined by Breman et al [34]. Although overall health insurance coverage is low, FHHs reported even lower levels of insurance coverage than MHHs. Instalmental payment which is a form of cash payment but with the ability of an extended repayment period enables households to absorb the shock of seeking care and was reported by both MHHs and FHHs in the FGDs. This was reported mainly in the rural area which suggests that there is a form of cooperation between health care providers and households in the rural areas.

Although MHHs incurred higher health care expenditures, FHHs experienced a higher health cost burden across all socio-economic groups, but particularly for the two poorest groups. Based on the FGDs, women attributed ill-health to their socio-economic context. Gendered norms around masculinity were likely to have prevented men from speaking of their health whereas women spoke more freely of their illness experiences. Considering that primary health centres are located in the rural areas and that the study population is predominantly rural, primary health centres could have provided FHHs better access to best possible health- care for the treatment of their illness at affordable cost, but clearly not many households used their services as can be seen in the FGDs. While FHHs reported higher cost burdens than MHHs, both cost burdens were catastrophic going by the definition of catastrophic expenditure by Breman et al. [34]. In addition, FHHs reported higher levels of untreated morbidity than MHHs. This depicts a picture which shows that the most vulnerable to catastrophic expenditures also do not seek care with affordability as the reason for not seeking care.

For those that sought care, there was a high utilisation of PMVs. Although not statistically significant in the quantitative analysis, as can be seen in the FGDs, the utilisation of PMVs and private hospitals is as a result of perceptions of inefficiency and ineffectiveness in primary health centre and public hospitals. The utilisation of PMVs and private hospitals in turn has their own implications on health outcomes and cost burdens. While private hospitals are more effective than primary health centres and public hospitals, they are more expensive and hence households incur high cost burdens [20]. On the other hand, PMVs which are unregulated are cheaper due to ineffective care they provide; they sell drugs based on demand and not based on prescription [35]. This has serious implications on health outcomes and may result in household seeking more effective care as sickness persists thereby incurring more cost. Similar patterns of treatment-seeking have been observed in other parts of Africa and Asia where there is a progression from affordable and less effective health care providers to more effective unaffordable providers as illness persists [34], [36]–[40]. This has important consequences for FHHs due to their higher morbidity especially since previous research has found that these low-level health care providers like PMVs are unregulated, likely to offer very low-quality treatment and do not have trained personnel [41].

To cope with health care costs, both MHHs and FHHs discussed drawing on savings, sale of assets and borrowing as the main strategies employed. These were picked up partly in the survey and largely in the FGDs. The important role of borrowing from informal or social network sources (friends, neighbours, relatives) as a coping strategy has been identified elsewhere [42]–[45] and this was mainly reported by both MHHs and FHHs. Borrowing is much more readily available to households which have fairly well-off friends and who are less likely to hold-up repayment [3], [44], [46], [47]. Although borrowing from informal structures is considered a low risk tactic, borrowing from semi-formal structures like money lenders and associations as the most unfavourable source of funds and can have negative implications for household’s economic and social position due to the high interest rates charged particularly if debts are not repaid on time. This is has important implications for treatment seeking and affordability particularly for FHHs in this study due to their socio-economic status.

An important finding relates to the strategies employed to pay back health care related debt. Although both MHHs and FHHs reported arduous strategies (e.g. household heads or children leaving home to work on the farms of the creditor), the desperation of women and particularly widows who reported working on construction sites to eke out a living in order to repay debt and also cutting back on consumption is concerning. This has dire consequences for their health status and in-turn contributes to a high illness burden which will require care hence triggering the “medical poverty trap” as inferred by Whitehead et al [48].

The plight of widows in Enugu State (same location of the present study) has been previously highlighted by Ugwu ([49] p:1622), who argued that “…widows have particularly low social and economic status. As a result of loss of husbands to AIDS or ill-health they have no inheritance rights to productive resources such as land, farm inputs, cash crops and family assets e.g. processing machines etc. In most cases, they are victims of seclusion, isolation, inhuman social treatments from their husband’s relatives and the community. These have implications for household food security, family cohesion and sustainability of rural livelihoods.” These findings offer an explanation to the results obtained from FHHs in this study.

## Policy Implications

This study provides evidence that efforts to protect the poor are critical from the adverse impact of OOPs and that positive measures to improve household’s socio-economic status are necessary. “Reducing or removing all user fees in government-run health care facilities would be a constructive move towards protecting households from high costs of illness” and “such an approach still requires extra resources to meet the likely rise in demand for health care and to guarantee that the quality of care is improved and maintained” ([44] p:681). These changes have to be carefully planned and implemented to prevent negative implications [50].

At the same time, it is suggested that an improvement in the public health care system in terms of quality of care and availability of care will encourage people from seeking care in the public sector and protect them from incurring higher costs and ineffective care in the private sector, or failing to seek treatment altogether. Primary health centres need to be improved in terms of resources and quality of care in order to improve the public perception and be the first point of care. Physical access can be achieved through the building of primary health centres in areas that are presently underserved. Properly trained and government paid community-based health workers may well also be used to increase access to quality health- care services. Unless this occurs, household will continue to seek care at PMVs.

It is to be anticipated that any interventions to improve health seeking for the poor have to engage the low level providers (primary health centres and PMVs). These providers are ever-present in all crannies of the country and form the major source of drugs, advice, and other consultancy services for majority of the population. If efforts to regulate PMVs are successful with respect to quality of care and the provision of good quality drugs, an improved access to quality care for especially the poorest households will be ensured [35].

Breman et al. [34] suggests that even if health care services are enhanced, they cannot guard households from all illness costs. He recommends that health policy research and debates ought to be broadened to consist of interventions beyond the health sector; interventions focused on enhancing the livelihoods of households, that save the poor from harm and increase their incomes. This study supports this ideology and suggests interventions such as supporting micro finance schemes that provide finance for small and medium-scale enterprises and provide avenues to encourage people to save weekly or monthly. Schemes which focus on FHHs and widows in particular are critical for ensuring access to health care and protection from catastrophic costs.

Onah suggests that since every female is a potential widow (67% of women outlive their husbands in a Nigeria), a call for strides towards the elimination of harmful widowhood practices in Nigeria is necessary [51]. State and federal enactments that protect women from these practices need to be established and where established must be enforced to ensure social protection of the most vulnerable of this population (widows and siblings). This study supports this call for full government involvement in the financial protection and empowerment of women especially widows.

## Limitations

The cross-sectional household survey questionnaire did not take into account inpatient and outpatient distinctions in the economic cost of seeking care. It also did not factor in the peculiarities of polygamous households which arguably have implications for access to resources and decision making.

Although the study intended to provide a breakdown of OOPs and identify the contributions of the different components (e.g. transport, drugs, consultancy costs), it was not possible to establish this because of difficulties in the interview process where household members were not able to recall this information. This is an important area for further research.

There are also the limitations posed by interviewer bias and problems associated with respondents feeling comfortable and disclosing all information in the household surveys. For instance, sale of assets was not mentioned as a coping strategy in the household surveys but was spoken of by the majority of participants in the FGDs.
